# Extrapelvic endometriosis in abdominal wall scar and PPAR gamma expression: A case report

**DOI:** 10.1016/j.ijscr.2018.10.026

**Published:** 2018-10-24

**Authors:** Achmad Kemal Harzif, Melisa Silvia, Ana Mariana, Lydia Olivia, Bara Tracy Lovita, Budi Wiweko

**Affiliations:** a Division of Reproductive Endocrinology and Infertility, Department of Obstetric and Gynecology, Faculty of Medicine, Universitas Indonesia, Dr.Cipto Mangunkusumo General Hospital, Jakarta, Indonesia; b Department of Obstetric and Gynecology, Faculty of Medicine, Universitas Indonesia, Dr.Cipto Mangunkusumo General Hospital, Jakarta, Indonesia; c Indonesian Reproductive Medicine Research and Training Center (INA- REPROMED) of Faculty of Medicine, Univesity of Indonesia, Ciptomangunkusumo Hospital Jakarta, Indonesia

**Keywords:** Extrapelvic endometriosis, Abdomen wall, PPAR, R gamma, Case report

## Abstract

Introduction: Endometriosis is a frequent gynecological disorder. Its findings on the abdominal wall are rare cases, usually reported with a history of former surgery. The activation of PPARy expression could be one of alternative non-hormonal treatment for endometriosis.

Presentation of case: A patient, 34 years old, suspected of endometriosis since 5 years ago because of pain during menstruation. Since the last 4 months, she had worsening of dysmenorrhea and palpable lumps during menses. She had previously performed cesarean section surgeries 3 times and a laparotomy cystectomy due to left endometriosis cyst. From ultrasound examination, on the abdominal wall (submusculus) there is a firmly defined hypoechoic mass, sized 32 × 10 × 15 mm, derived from the mass of endometriosis.

Discussion: The endometriosis findings on the abdominal wall are rare cases. Symptoms usually are cyclic pain which is also accompanied by a palpable lump. A meta-analysis of 16 studies concluded that sensory excitatory threshold and pain tolerance approached the lowest value shortly before and during menstruation. In humans, administration of PPARy agonists reduced pain symptoms. Counseling to patients is given about the possibility of recurrence.

Conclusion: PPAR expressivity assessment has not been used as a target for endometriosis therapy. Further studies separating epithelial tissue and stroma can be performed to prove the role of PPARγ in the pathogenesis of endometriosis.

## Highlights

• The extrapelvic abdominal wall endometriosis is a rare cases.
• The ectopic abdominal wall endometriosis express higher PPARγ activity compared to eutopic endometrium.
• In humans, administration of PPARy agonists reduced pain symptoms.
• Counseling to patients is given about the possibility of recurrence.

## 1 Introduction

Endometriosis is a frequent gynecological disorder with an 8%–15% prevalence. The extrapelvic implantation of endometrial tissue is found in various organs and systems, the findings on the abdominal wall are rare cases, usually reported with a history of former surgery. A non-hormonal treatment that can be used for long period without significant side effects and with no impact on fertility is urgently needed for endometriosis patients. The activation of PPARy receptor expression could be one of alternative non-hormonal treatment for endometriosis. Recently, as inhibition of angiogenesis, PPAR-γ ligands in addition to their established role as tumor cell cycle modulators could be implicated in future strategies for cancer treatment.

This study’s introduction has been reported in line with the SCARE criteria [1].

## 2 Presentation of case

**Fig. 1:**
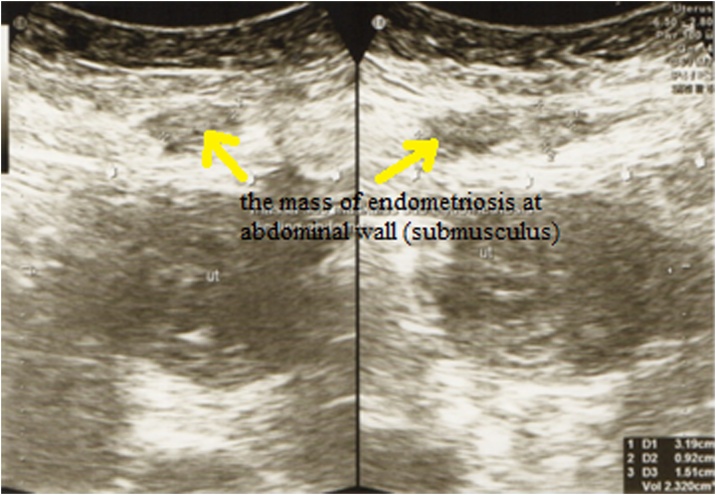
Ultrasound examination results.

**Fig. 2:**
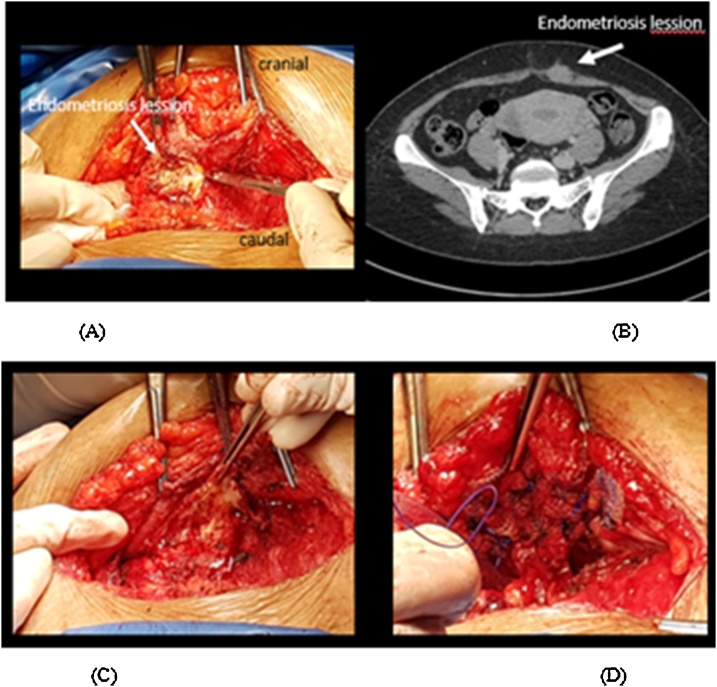
Endometriosis lesions during excision procedure.

**Fig. 3:**
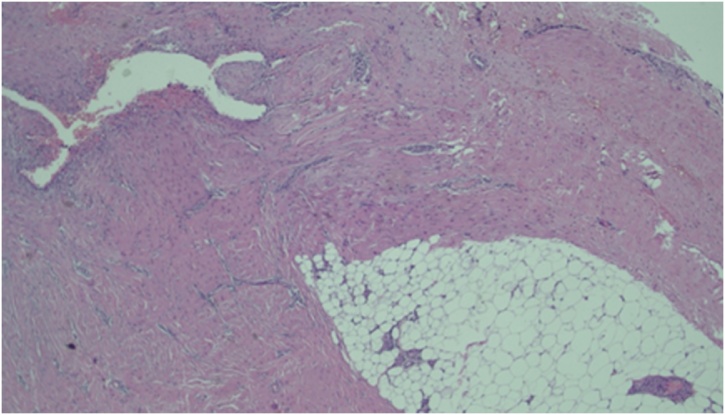
Histological appearance.

A patient, 34 years old, parity 3 came with painful abdominal pain during menstruation (VAS 6–8) which getting worst since 4 months before admission. Pain is felt during first day of period until the third day (since last 5 years). Palpable lump in the abdomen during pain. Previously had been taking drugs such as analgesic and progesterone synthetic but pain was not relieved. Her LMP was January 7^th^, 2017. Patients had done ultrasound and CT scan in RSUD Serang revealed an abdominal wall endometriosis (Fig. 1, Fig. 2, Fig. 3).

Patients had previously performed cesarean section 3 times and 1 laparotomy for indication of left endometriosis cyst. She got menarche at age 12 years, regularly with 30-day cycle, duration 4–5 days, change pads 2 - 3x days, menstrual pain was present. Married 1 time.

On physical examination, the abdomen is seen to be supple, palpable lumps of 5 × 5 cm between mid-central and symphysis, limited mobility, positive bowel sounds. Gynecological status, vulva and urethra are calm. The inner examination within normal limits.

The result of ultrasound was a retroflexed uterus of normal shape and size; It appears that some hypoechoic masses are firmly derived from intra-mural fibroid. Regular endometrium with 5 mm thick. On the abdominal wall (submusculus) there is a firmly defined hypoechoic mass, size 32 × 10 × 15 mm derived from the endometriosis. Conclusion: the mass of endometriosis at abdominal wall with adnexal adhesions.

Laboratory test obtained Hb 13.6 g / Dl, Ht 35.2%, Leukosit 5730 / Ul, platelet 258,000 / Ul. PT / APTT: 1x / 1x, Ureum 16 mg / Dl, creatinine 0.5 mg / dl, SGOT / SGPT: 23/28 u / L. Albumin 4.04 g / Dl. GDS 111 mg / Dl. Na: 146 / K: 4.75 / Cl: 104. Ca-125: 23, AMH: 1.5 ng/Ml

Patients diagnosed with abdominal pain due to endometriosis of the abdominal wall. Laparotomy excision of mass and mesh placement was performed in the subfascial layer.

Histopathology examination shows glands and endometrial stroma between the connective tissue. Tubular glands are partially widened, coated with columnar epithelium. Cellular stroma with swollen areas. There are some lymphocytes (inflamatory reaction) and hemosiderofags. Histologically corresponds to external endometriosis of the abdominal wall.

A sample of the abdominal endometriosis and eutopic endometrium was sent for PCR examination of PPARγ mRNA expression. Each samples was measured three times and the median expression of PPARγ mRNA expression is 21.85 ng/μl for eutopic endometrium and 26.84 ng/μl for abdominal wall endometriosis.

Treatment is followed by 2 mg of oral 2 mg of oral progestin twice daily for the long term. Counseling is given to the patient about the possibility of recurrence of endometriosis lesions. Observation for 3 months, no complaints have been received in patients.

## 3 Discussion

Endometriosis is a frequent gynecological disorder [2]. The findings on the abdominal wall are rare cases, usually reported with a history of former surgery [2]. Symptoms usually are cyclic pain which is also accompanied by a palpable lump [2]. In patients suspected of endometriosis since 5 years ago due to pain during periods, since the last 4 months the pain felt heavy and palpable lumps that hardened during the pain.

The mechanism of the pain in endometriosis are direct and indirect effects on focal bleeding originating from endometriosis implants, the effects of proinflammatory cytokines present in the peritoneal cavity and irritation or direct infiltration of innervation in the pelvic cavity.^3^ A meta-analysis of 16 studies concluded that sensory excitatory threshold and pain tolerance approached the lowest value shortly before and during menstruation [3].

In endometriosis, serum Ca-125 levels may be elevated mildly [4]. However, it is not a specific marker because it also increases in other inflammatory conditions, but can be used as a marker to see a recurrence or response to therapy given [[5], [6], [7]].

Non hormonal treatments such as NSAID do not interfere with fertility, but long-time treatment with such agents may pose significant side effects. A non-hormonal treatment that can be used for long period without significant side effects and with no impact on fertility is urgently needed for endometriosis patients who wish to conceive [8].

In this case we found that the abdominal wall endometriosis express PPARγ activity and its value is higher compared to eutopic endometrium. Activation of PPARy reduces aromatase, thus reducing the production of estrogen in endometriosis lesions and reducing the proliferation of endometriosis lesions in vitro [9]. In humans, administration of PPARy agonists reduced pain symptoms. It has been reported that increased PPARy expression in peritoneal lesions reduced symtomps in endometriosis patients, especially pelvic pain, dysmenorrhea, and dyspareunia [10,11]. Expression of PPARy receptor in endometriosis tissue may be useful to study its role in the pathogenesis of endometriosis and as a predictor in determining the response to therapy with PPARy ligands [11]. Research is needed to evaluate different PPARy expression in endometriosis case and compare to normal control.

Counseling to patients is given about the possibility of recurrence. Rarely occurs recurrence, if it occurs within the first year and possibly associated with inadequate excision [2,4].

## 4 Conclusion

Extrapelvic endometriosis is a rare case. In this case, endometriosis is obtained in the abdominal wall scar tissue. The findings revealed that there were no significant differences on PPARγ expression of endometrium between endometriosis and no endometriosis group. PPAR expressivity assessment has not been used as a target for endometriosis therapy. Further studies separating epithelial tissue and stroma can be performed to prove the role of PPARγ in the pathogenesis of endometriosis.

## Conflicts of interest

There is no conflict of interest in this study.

## Funding

There is no such involvement sponsor in this study.

## Ethical approval

This study was approved by the Ethics Committee of Faculty of Medicine, Universitas Indonesia, on October 2014 (reference number: 733/UN2.F1/ETIK/2014).

## Consent

Written informed consent was obtained from the patient for publication of this case report and accompanying images. A copy of the written consent is available for review by the Editor-in-Chief of this journal on request.

## Author contribution

Achmad Kemal Harzif, MD, OG (REI) who has study concept, resources, validation and supervision.

Melisa Silvia, MD and Ana Mariana, MD who have data collection.

Lydia Olivia, MD, OG who has data analysis and writing paper.

Bara Tracy Lovita, MD who has editing and prepare the manuscript.

Budi Wiweko, MD, OG (REI) who has review manuscript and supervision.

## Registration of research studies

Researchregistry4468.

## Guarantor

Achmad Kemal Harzif, MD, OG (REI).

Provenance and peer review

Not commissioned externally peer reviewed.
